# To Assess the Variation in the Definitions Used to Describe the Stages of Acute Charcot Neuro‐Osteoarthropathy (Diagnosis, Remission and Relapse): A Systematic Review

**DOI:** 10.1002/jfa2.70106

**Published:** 2025-12-08

**Authors:** Dipen Patel, Catherine Gooday, Katie Gray, Ketan Dhatariya

**Affiliations:** ^1^ Norwich Medical School University of East Anglia Norwich UK; ^2^ Elsie Bertram Diabetes Centre Norfolk and Norwich University Hospitals NHS Foundation Trust Norwich UK; ^3^ Derbyshire Community Health Services NHS Chesterfield UK

**Keywords:** charcot neuro‐osteoarthropathy, diagnosis, relapse, remission

## Abstract

**Background and Aims:**

In 2023 the International Working Group of the Diabetic Foot (IWGDF) published definitions for the diagnosis, remission and relapse of active Charcot neuro‐osteoarthropathy (CN). Until 2023 authors had used varied definitions to identify the stages of CN making comparisons difficult. The aim of the current study was to illustrate variation in definitions used before 2023 and to encourage consistent terminology in future publications.

**Methods:**

Searches were conducted in OVID, Pubmed and CINAHL. Studies were included if they had a minimum of 10 participants and reported the outcomes of CN in conservative or pharmacological treatments in people with diabetes and CN. Included study designs were randomised controlled trials, cohort studies, non‐randomised controlled trials, case‐controls and case series. We excluded studies of surgical treatment of CN. Data collected included location of the study, study design and population. Definitions of each stage were compared to the IWGDF definitions through a scoring system.

**Results:**

We screened 1758 titles, 143 abstracts and 63 full texts. We included 37 studies from 20 different countries. Scores comparing the studies' definitions to the published IWGDF guidelines for definitions ranged from a similarity of 17%–100%. Six studies scored 100% with 31 studies missing aspects of the IWGDF definitions.

**Conclusion:**

Authors should use standardised definitions of the stages of CN to allow comparisons between studies to be accurately made. More work is needed to assess the variation in terminology within studies with CN treated surgically.

**Abbreviations**
CN: Charcot Neuro‐osteoarthropathy
CT: Computed Tomography
IWGDF: International Working Group of the Diabetic Foot
MRI: Magnetic Resonance Imaging
MTP: Metatarsophalangeal
T1DM: Type 1 Diabetes Mellitus
T2DM: Type 2 Diabetes Mellitus
TMT: Tarsometatarsal

## 1 Introduction

Until the recently published IWGDF systematic review and guidelines papers (2023) there had been no internationally agreed definitions for the stages of Charcot neuro‐osteoarthropathy (CN); diagnosis, remission and relapse [1, 2]. Therefore, individual clinical and research teams have devised their own definitions. The process of individual teams developing these recommendations has varied and is not always based on robust methodological review of the evidence. A pragmatic approach such as the availability and access to diagnostic equipment such as infrared thermometry or radiological imaging can be the rationale behind why one team uses a different approach to CN diagnosis, remission and relapse. Authors differ in their hierarchy of values, with some regarding clinical criteria more valuable than radiology, with others feeling the reverse. These differences may be reflected in the way they order and interpret the results of clinical examination, laboratory, radiological or thermography tests for CN.

The inconsistencies in the definitions used to identify the three key stages of CN; diagnosis, remission and relapse, could in part contribute to the variations in outcome reported for the condition. Case series report treatment times ranging from 28 to 150 days, ulceration rates ranging from 21.1% to 72.3% and amputation rates ranging from 1.7% to 19.1% [3, 4, 5, 6, 7]. The lack of standardised terminology for CN means that clinical teams can find it difficult to decide when to start or stop treatment, potentially leading to under or overtreatment of CN and this can negatively impact on the quality of life and outcomes of people with CN.

### 1.1 Diagnosis

When it comes to a definition of diagnosis, the clinical criteria are the signs and symptoms that individuals experience. This is having a hot, red, swollen foot with temperature differences of at least 2°C between the affected and the unaffected foot. The radiological criteria used in previous studies can show the classification of the CN according to the Eichenholtz classification system [8, 9]. Different studies have used a varying combination of clinical and radiological criteria for the diagnosis of CN [3, 4, 5, 6, 7, 10].

### 1.2 Remission

Current definitions of remission include a mix of an improvement of clinical and radiological criteria such as having temperature differences of below 2°C between the affected foot and unaffected foot as well as improving sclerosis on X‐rays. In addition to this, in some studies, the clinical decision to discontinue treatment such as cast walkers would be used for remission criteria [4, 6, 7, 10].

### 1.3 Relapse

Similar criteria to the definition of diagnosis were used in the definition of relapse across research studies [4, 5, 6, 11, 12]. Some studies would use this in combination with a timeframe from the last treatment of CN.

The lack of a standardised definition could mean studies results are less valid but more importantly make it difficult to compare findings between studies.

We aimed to examine and evaluate the published definitions, in studies between 1993–2023, used to describe the three phases of CN (diagnosis, remission and relapse) and compare them to the agreed 2023 definitions published by the International Working Group of the Diabetic Foot (IWGDF). We aimed to demonstrate the variability in the definitions used in research prior to the publication of the IWGDF definitions.

## 2 Methods

Searches were undertaken in OVID, Pubmed and CINAHL to find potential eligible studies. The searches were restricted to English language studies from 1993–2023.

### 2.1 Search Strategy

#### 2.1.1 Inclusion and Exclusion Criteria

Studies were included if they reported foot and/or ankle outcome of diabetes related CN, had a minimum of 10 participants, in studies reporting the outcomes from conservative or pharmacological management of CN. The study designs that were included were randomised controlled trials, cohort studies, non‐randomised controlled trials, case‐control studies and case series. The studies included were between 1993 and 2023.

Studies were excluded if the main focus was not CN, the CN was not related to diabetes, the CN was at a different anatomical location (i.e., not the foot) or the studies were focused on surgical interventions. The study designs that were excluded are systematic reviews, meta‐analyses, narrative reviews, epidemiological studies, case series or reports with < 10 participants, audits and conference abstracts.

### 2.2 Screening

Screening occurred in three stages; title, abstract and full text. Two researchers (DP, CG) worked independently and screened at each stage against the inclusion and exclusion for the review. The same two researchers settled any conflicts together after each stage. A third independent researcher (KD) settled any conflicts that were still present. Inter‐rater agreement was calculated at each stage of the screening process by the number of papers agreed on divided by the total number of papers screened.

### 2.3 Data Extraction

Data on study characteristics, participant characteristics and definitions of diagnosis, remission and relapse were extracted. The International Working Group of the Diabetic Foot (IWGDF) published guidelines in 2023 regarding the recommendations of diagnosis, identification of remission, treatment and relapse [1]. The data was then analysed by comparing the quoted definitions to assess the similarities and differences in the definitions of diagnosis, remission and relapse to those from the IWGDF.

A scoring system was designed in order to quantify the similarities and differences of the study definitions used in the included studies to the IWGDF agreed definitions [1]. The scoring system is summarised in Table 1.

**TABLE 1:** Definition Scoring System used to assess the quality of the key stages of Charcot neuro‐osteoarthropathy [1].

Term	Score			
	0	1	2	3
Diagnosis	No definition/missed key aspects	One of the following included: Clinical findings Assessed radiologically Temperature difference using infrared thermometry	Two of the following included: Clinical findings Assessed radiologically Temperature difference using infrared thermometry	All the following included: Clinical findings Assessed radiologically Temperature difference using infrared thermometry
Remission	No definition/missed key aspects	One of the following included: Absence of clinical findings Assessed radiologically (if present‐ consolidation of fractures) Temperature difference using infrared thermometry	Two of the following included: Absence of clinical findings Assessed radiologically (if present‐ consolidation of fractures) Temperature difference using infrared thermometry	All the following included: Absence of clinical findings Assessed radiologically (if present‐ consolidation of fractures) Temperature difference using infrared thermometry
Relapse	No definition/missed key aspects	One of the following included: Return of clinical findings in ipsilateral foot Assessed radiologically after remission Temperature difference using infrared thermometry	Two of the following included: Return of clinical findings in ipsilateral foot Assessed radiologically after remission Temperature difference using infrared thermometry	All the following included: Return of clinical findings in ipsilateral foot Assessed radiologically after remission Temperature difference using infrared thermometry

Each study was scored out of 3 for each definition that was assessed relevant for the individual studies included in this review. Each study scored between 0 (no definition/missed key aspects in all three stages) to a maximum score of 9 (all definitions in line with the IWGDF 2023 guidelines.

### 2.4 Ethical Permission

Ethical permissions were not required for this work because no personal or identifiable materials were used.

## 3 Results

### 3.1 Search Results

**FIGURE 1:**
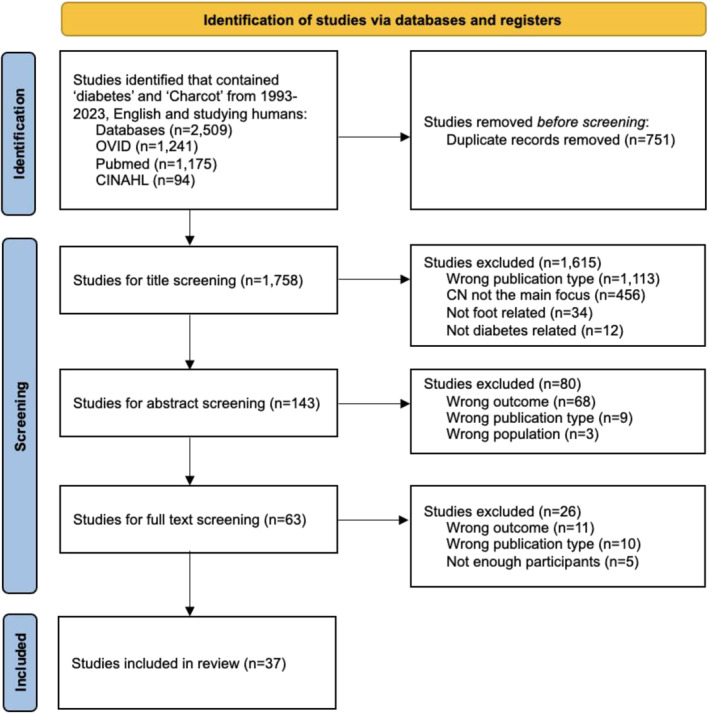
PRISMA flow diagram.

After the removal of duplicates, we identified 1205 papers (Figure 1) and excluded 1065 papers during title screening. During abstract screening we excluded 95/140 papers, most exclusions concerned expert reviews, papers describing other aspects of care and conference abstracts.

There were 37 studies (Table 2) that met the inclusion and exclusion criteria. There were 23 retrospective papers and 14 prospective papers. Of these 27 were cohort studies, 7 randomised controlled trials, 1 non‐randomised controlled trial, 1 case‐control study and 1 case series.

**TABLE 2:** List of included studies.

Author	Reference	Title
Studies looking at Charcot neuro‐osteoarthropathy monitoring		
Armstrong & Lavery (1997)	[10]	Monitoring healing of acute Charcot's arthropathy with infrared dermal thermometry
Moura‐Neto et al. (2012)	[13]	Charcot foot: Skin temperature as a good clinical parameter for predicting disease outcome
Wanzou et al. (2019)	[14]	Charcot arthropathy of the diabetic foot in a sub‐Saharan tertiary hospital: a cross‐sectional study
Studies looking at Charcot neuro‐osteoarthropathy outcomes		
Al‐Rubeaan et al. (2020)	[15]	Characteristics of patients with Charcot's arthropathy and its complications in the Saudi diabetic population: A Cross‐Sectional study
Armstrong et al. (1997)	[11]	The natural history of acute Charcot's arthropathy in a diabetic foot speciality clinic
Berli et al. (2021)	[3]	The “Balgrist score” for evaluation of Charcot foot: a predictive value for duration of off‐loading treatment
Chantelau (2005)	[16]	The perils of procrastination: Effects of early versus. delayed detection and treatment of incipient Charcot fracture
Chantelau & Schnabel (1997)	[17]	Palliative radiotherapy for acute Charcot's arthropathy with infrared dermal thermometry
Chaudhary et al. (2019)	[18]	Mortality in Asian Indians with Charcot's neuroarthropathy: a Nested cohort prospective study
Christensen et al. (2012)	[12]	Duration of off‐loading and recurrence rate in Charcot osteo‐arthropathy treated with less restrictive regimen with removable walker
de Souza (2008)	[7]	Charcot arthropathy and immobilisation in a weight‐bearing total contact cast
Doria et al. (2018)	[19]	Short‐term foot complications in Charcot neuroarthropathy: a Retrospective study in tertiary care in Spain
Engberg et al. (2022)	[20]	The clinical course and mortality of persons with diabetic Charcot foot
Fabrin et al. (2000)	[6]	Long‐term follow‐up in diabetic Charcot feet with spontaneous onset
Gratwohl et al. (2022)	[5]	Long‐term follow‐up of conservative treatment of Charcot feet
Griffiths et al. (2021)	[21]	Duration of total contact casting for resolution of acute Charcot foot: a retrospective cohort study
Jansen et al. (2018)	[22]	Mortality and complications after treatment of acute diabetic Charcot foot
Leung et al. (2013)	[23]	Charcot foot in a Hong Kong Chinese diabetic population
Myerson et al. (1994)	[24]	Management of midfoot diabetic neuroarthropathy
O’Loughlin et al. (2016)	[25]	Diabetic Charcot neuroarthropathy: Prevalence, demographics and outcome in a regional referral centre
Osterhoff et al. (2013)	[4]	Recurrence of acute Charcot neuropathic osteoarthropathy after conservative treatment
Pakarinen et al. (2002)	[26]	Charcot arthropathy of diabetic foot. Current concepts and review of 36 cases
Pakarinen et al. (2013)	[27]	Effect of immobilisation, off‐loading and zoledronic acid on bone mineral density in patients with acute Charcot neuroarthropathy: A prospective randomised trial
Saltzman et al. (2005)	[28]	How effective is intensive nonoperative initial treatment of patients with diabetes and Charcot arthropathy of the feet?
Sebastian et al. (2019)	[29]	Clinical features, radiological characteristics and offloading modalities in stage 0 acute Charcot neuroarthropathy: A single centre experience from South India
Sinacore (1998)	[30]	Acute Charcot arthropathy in patients with diabetes mellitus: Healing times by foot location
Stark et al. (2016)	[31]	5 years retrospective follow‐up of new cases of Charcot neuropathy‐ A single centre experience
Thewjitcharoen et al. (2018)	[32]	Salient features and outcomes of Charcot foot – An often‐overlooked diabetic complication: A 17‐year‐experience at a diabetic centre in Bangkok
Verity et al. (2008)	[33]	Treatment of Charcot foot and ankle with a prefabricated removable walker brace and custom insole
Visan et al. (2012)	[34]	The role of the walker in the early treatment of Charcot foot
Wukich et al. (2011)	[35]	The consequence of complacency: Managing the effects of unrecognised Charcot feet
Studies looking at Charcot neuro‐osteoarthropathy outcomes with pharmacological treatment		
Bem et al. (2006)	[36]	Intranasal calcitonin in the treatment of acute Charcot neuroarthropathy: a Randomised controlled trial
Busch‐Westbroek et al. (2018)	[37]	Effect of single dose of RANKL antibody treatment on acute Charcot neuroosteoarthropathy of the foot
Jude et al. (2001)	[38]	Bisphosphonates in the treatment of Charcot neuropathy: A double‐blind randomised controlled trial
Pakarinen et al. (2011)	[39]	The effect of zoledronic acid on the clinical resolution of Charcot neuroarthropathy: a Pilot randomised controlled trial
Petrova et al. (2021)	[40]	Effect of recombinant human parathyroid hormone (1–84) on resolution of active charcot neuro‐osteoarthropathy in diabetes: A randomised, double‐blind, placebo‐controlled study
Pitocco et al. (2005)	[41]	Six‐month treatment with alendronate in acute Charcot neuroarthropathy: a Randomised controlled trial

Inter‐rater agreement was calculated at each stage of screening. It was 88.8% (1561/1758) at title screening, 81.8% (117/143) at abstract screening and 81.0% (51/63) at full text screening.

Of the 37 included papers, 7 were from the United States of America, 4 from Denmark, 3 each from the United Kingdom, Switzerland and Finland, 2 each from India and Germany, 1 each from Italy, Thailand, Romania, Canada, Brazil, Uganda, Saudi Arabia, Australia, Czech Republic, the Netherlands, China, Ireland and Spain.

### 3.2 Participant Characteristics

There were 2778 participants across the 37 studies with 2386 CN feet. Sex was reported in 32 studies; there were 1563 (65.0%) male participants and 841 (35.0%) female participants. The mean age of participants was reported in 29 studies, with a range between 51.3 and 62.5 years 26 studies reported the types of diabetes: 382 type 1 diabetes (T1DM) and 887 type 2 diabetes (T2DM). The reported mean duration of both T1DM and T2DM ranged between 7.0 and 34.4 years.

There were 17 studies that required a definition of diagnosis only, 12 studies required a definition of diagnosis and remission and eight studies required a definition of all three (diagnosis, remission and relapse). Table 3 summarises the total each study scored according to the scoring system.

**TABLE 3:** Total Score of all definitions (diagnosis, remission, relapse) in each included study.

Papers	Total score	Out of	Percentage (%)
Monitoring studies			
Armstrong & Lavery (1997)	6	6	100
Moura‐Neto et al. (2012)	5	9	56
Wanzou et al. (2019)	2	3	67
Outcome studies			
Al‐Rubeaan et al. (2020)	2	3	67
Armstrong et al. (1997)	5	9	56
Berli et al. (2021)	2	3	67
Chantelau (2005)	4	6	67
Chantelau & Schnabel (1997)	4	6	67
Chaudhary et al. (2019)	3	3	100
Christensen et al. (2012)	8	9	89
De Souza (2008)	1	6	17
Doria et al. (2018)	3	3	100
Engberg et al. (2022)	3	3	100
Fabrin et al. (2000)	4	9	44
Gratwohl et al. (2022)	4	6	67
Griffiths et al. (2021)	5	6	83
Jansen et al. (2018)	6	9	67
Leung et al. (2013)	2	3	67
Myerson et al. (1994)	2	6	33
O’Loughlin et al. (2016)	1	3	33
Osterhoff et al. (2013)	6	9	67
Pakarinen et al. (2002)	2	3	67
Pakarinen et al. (2013)	5	6	83
Saltzman et al. (2005)	2	6	33
Sebastian et al. (2019)	2	3	67
Sinacore (1998)	4	9	44
Stark et al. (2016)	2	6	33
Thewjitcharoen et al. (2018)	1	3	33
Verity et al. (2008)	2	6	33
Visan et al. (2012)	4	6	67
Wukich et al. (2011)	2	3	67
Outcomes studies with pharmacological treatment			
Bem et al. (2006)	3	3	100
Busch‐Westbroek et al. (2018)	3	6	50
Jude et al. (2001)	3	3	100
Pakarinen et al. (2011)	7	9	78
Petrova et al. (2021)	2	6	33
Pitocco et al. (2005)	2	3	67

Eight studies used the Sanders and Frykberg classification system, 10 used the Eichenholtz classification system and five used the Brodsky classification system [8, 9, 42, 43].

### 3.3 Definitions

#### 3.3.1 Assessment of Definitions Used to Describe the Diagnosis of Charcot Neuro‐Osteoarthropathy

Using the scoring system described in Table 1, all 37 studies were deemed to be eligible for the assessment of the quality of the definition of the diagnosis of CN. Of these 37 included studies, four scored 0/3, four scored 1/3, 18 scored 2/3 and 11 scored 3/3 on the scoring system. There were 31 studies that included clinical findings as a part of their definition of diagnosis.

There were 23 studies that reported using radiological methods for the definition of diagnosis. From these, 15 studies included using X‐rays, eight studies included using MRIs, seven studies included using other radiological types (including CT and bone scintigraphy) and 11 studies did not specify which radiological method was used. Nine studies reported using multiple radiological methods.

There were 13 studies that included the use of infrared thermometry to measure temperature differences. 10 of these studies reported the cut off temperature difference for the criteria of diagnosis, all of which reported using > 2°C.

### 3.4 Assessment of Definitions Used to Describe Remission of Charcot Neuro‐Osteoarthropathy

Of the 37 studies included, 21 studies were deemed to be eligible for the assessment of the quality of the definition of the remission of CN. Of these 21, two scored 0/3, five scored 1/3, ten scored 2/3 and four scored 3/3 on the scoring system. 13 studies included clinical findings as a part of their definition of diagnosis.

Thirteen studies reported using radiological methods for the definition of remission. From these, three studies included using X‐rays, three studies included using MRIs, two studies included using other types (both used bone scintigraphy) and eight studies did not specify which radiological method was used. Two studies reported using multiple radiological methods.

There were 11 studies that included the use of infrared thermometry to measure temperature differences. All of these studies reported the cut off temperature difference which varied; five studies used < 2°C, two studies used < 1°C, two studies used 1–2°C, one study used < 1.5° C.

### 3.5 Assessment of Definitions Used to Describe the Relapse of Charcot Neuro‐Osteoarthropathy

Eight studies were deemed to be eligible for the assessment of the quality of the definition of the relapse of CN. Of these eight, four scored 1/3 and four scored 2/3 on the scoring system. Seven studies included clinical findings as a part of their definition of diagnosis.

Two studies reported using radiological methods for the definition of relapse. Both of these studies used X‐rays and MRIs as part of the criteria.

There were five studies that included the use of infrared thermometry to measure temperature differences. Four of these studies reported the cut off temperature difference, all of which reported using > 2°C.

## 4 Discussion

This study looked at the definitions used to define diagnosis, remission and relapse in active CN before 2023 and compared them to those recommended by the IWGDF. We found that there was a large variation between definitions across studies. Only six studies included all three aspects of their relevant definitions that were concordant with the IWGDF guidelines. The remaining 31 studies missed key aspects in their definitions of each stage of CN making it difficult to accurately compare the outcomes from these studies.

There were differences between studies within the key aspects of the definitions. We also noted there were differences in the radiological imaging modalities used. As the studies spanned from 1993–2023, differences in availability of different scans changed. Previously MRIs were not readily available so studies published earlier may not have used MRI scans in their definitions whereas more recent papers may be likely to include MRI as an option.

There were also differences between studies with the temperature differences reported between affected and unaffected feet. The most commonly reported temperature difference cutoff was 2°C, but the temperature differences ranged from 1 to 2°C.

Until the publication of the IWGDF guidelines, there had not been gold standard definitions for the stages of CN [1]. This has led to variation with individual clinical teams and researchers developing locally agreed criteria prior to 2023.

Varying definitions of the stages of CN can have research implications, these include, but are not limited to, making the comparison of outcomes between studies difficult, and having clinical implications, leading to under or over treatment. This would affect patient quality of life as over treating could lead to more time being spent immobilised and under treating could lead to deterioration of the CN, which could require surgical intervention and increased risk of complications such as amputation or mortality. Using universally agreed standardised definition of remission would ensure that there is a consistent approach to identifying the different stages of CN.

Despite an agreed definition being published, it may not always be feasible to apply this research into clinical practice. There are external factors that would affect this such as having additional training for clinicians, resources not being readily available such as infrared thermometry or different imaging modalities being more accessible than others.

The strengths of this paper include having strict inclusion criteria whilst covering a wide variety of study types, allowing a wider assessment to be made. We used three databases to conduct the search which minimises the risk of missing studies. The included studies are diverse representing 20 countries, have different populations and include authors from different clinical specialities which helps to increase the generalisability of the results.

As a part of the study design, we excluded surgical papers because surgical papers tend to focus on the management of chronic CN. Due to the retrospective nature of some of the studies included and insufficient descriptions of the stages of CN the assessment may not accurately reflect the definitions used in clinical practice. Some papers did score 0/3 if a definition was not explicitly stated. We believe this was an omission from the study manuscript rather than teams not locally agreed definitions. It should also be noted that the scoring system used to compare definitions was not validated, piloted or subjected to inter‐rater reliability. Additionally, the 2023 IWGDF definitions were also based on limited research as CN remains underexplored.

## 5 Conclusion

All studies reporting the outcomes of CN should now use the IWGDF criteria when defining diagnosis, remission and relapse of CN. Individual studies need to clearly state the clinical and radiological criteria used within the study for the key stages of CN and any deviation from the IWGDF recommendations explained and justified. The use of consistent terminology for diagnosis, remission and relapse is the first stage in a process to compare and benchmark the outcomes for CN, on a regional, national and international level. A better understanding of the outcomes will drive future research, improve clinical outcomes and the experiences of people who receive treatment for CN.

## Author Contributions

**Dipen Patel:** data curation, formal analysis, investigation, methodology, validation, writing – original draft, writing – review and editing. **Catherine Gooday:** conceptualization, data curation, formal analysis, investigation, methodology, project administration, resources, supervision, validation, writing – review and editing. **Katie Gray:** data curation, formal analysis, investigation, writing – review and editing. **Ketan Dhatariya:** conceptualization, data curation, formal analysis, methodology, project administration, resources, supervision, validation, writing – review and editing.

## Funding

The authors have nothing to report.

## Conflicts of Interest

The authors declare no conflicts of interest.

## Permission to Reproduce Material From Other Sources

Permission was granted by Dr. Catherine Gooday and Ms. Katie Gray to reproduce data from ‘Systematic review of techniques to monitor remission of acute Charcot neuroarthropathy in people with diabetes’.

## Data Availability

Data can be made available upon requested from the corresponding author.
